# Collecting family planning intentions and providing reproductive health information using a tablet-based video game in India

**DOI:** 10.12688/gatesopenres.12818.2

**Published:** 2018-09-07

**Authors:** Elena Bertozzi, Amelia Bertozzi-Villa, Praveen Kulkarni, Aparna Sridhar

**Affiliations:** 1 Department of Game Design & Development, Quinnipiac University, Hamden, CT, USA; 2 Institute for Disease Modeling, Bellevue, WA, USA; 3 Department of Community Medicine, JSS Medical College, JSS Academy of Higher Education and Research, Mysuru, Karnataka, India; 4 Department of Obstetrics and Gynecology, David Greffen School of Medicine, Los Angeles, CA, USA

**Keywords:** Family planning, games for health, sexuality, education, adolescents, reproductive health, videogame, India

## Abstract

**Background**: In response to a Grand Challenges in Global Health call for action to collect data about family planning intentions and increase the uptake of family planning methods in India, our team designed, developed, and piloted the *My Future Family* video game in Karnataka Province. The game educates adolescents about human sexuality and reproduction while asking players when they would like to achieve five important family planning milestones.  Participants were also asked to report who influences them the most when making family planning decisions.

**Methods**: Focus groups were conducted and the resulting data used to design the game which was iteratively tested and then piloted in 11 schools in rural and urban areas of southern India. Data was collected throughout gameplay and cross-checked with paper questionnaires.

**Results:** In August 2017, we successfully piloted the game with 382 adolescents and validated its efficacy both as an educational tool and as an innovative means of accurate data collection.

**Conclusion:** It has historically been problematic to gather accurate data about adolescents in India on this culturally sensitive topic for a variety of reasons. These include difficulties obtaining consent, developing appropriate survey methods, and framing questions in language that young people can understand. Our game met these challenges by working within a single school system with approval from senior administration, delivering information via a game environment which freed players from societal constraints, and communicating information via images and audio in addition to text in both English and Kannada (the local language).

## Introduction

Reproductive health education is crucial to overall health, female empowerment, and economic development worldwide. The situation in India is especially urgent due to longstanding difficulties in increasing the uptake of family planning methods, the persistence of early marriages, and failures of other methods of controlling population growth ^1^. Despite Prime Minister Modi’s efforts to encourage smaller families ^2^, and both internal interventions ^3^ and those from a variety of NGOs ^4^, progress has been slow ^5^. Furthermore, gaps in current survey methods (i.e. DHS ^6^) result in very little available data on what adolescents desire from a family planning perspective and whether or not they know how to achieve those goals.

Our aim in this study was to develop, pilot, and assess an innovative tool: a tablet-based game which provides adolescents with information about puberty, reproductive health, and family planning while collecting data. Data points include: desired family size, timing of important life events, and identity of principal influencers of these decisions. We specifically sought to address gaps in existing data by carefully designing the game-based survey methods ^7^. Our test population was adolescents (registered age 14–19) attending schools under JSS Mahavidyapeetha in the province of Karnataka, India. We sought to reach both female and male adolescents because previous studies have shown that family planning initiatives are more successful when both males and females are involved ^8^.

Results from gameplay indicated that students on average wanted 2 children, starting at age 26.8 (SD 3.59) for males and 25.4 (SD 3.44) for females. Students overwhelmingly wanted to delay childbirth until after the completion of education and the start of a career, and mostly wanted to restrict the number of children to one or two. Most importantly, we validated the use of a game to collect data and educate adolescents about a topic that is culturally sensitive and very important to future behavioral choices.

## Methods

### School selection

The JSS Mahavidyapeetha school system is a collection of grades 1–12 schools throughout Karnataka, India, with a focus on educating low-income students in both urban and rural areas. We used convenience sampling to select 11 of JSS’s 126 schools for this study.

### Focus groups

During game development, we conducted focus groups with parents, teachers, and students at the schools. Our aim was to understand what adolescents already knew about reproductive health and sexuality, what they wanted to know, and how teachers and parents preferred broaching the topic. A full list of questions asked in focus groups is included in Supplementary File 1. All interviews were conducted in Kannada. Focus groups were held at the JSS Medical School (a constituent college of JSS Academy of Higher Education and Research) in Mysore India in December 2016. Separate sessions (with ten participants each) were held with adolescent females, adolescent males, mothers, fathers, female teachers, and male teachers. During each session, we play-tested two different game prototypes to determine the feasibility of a game approach and if the participants were comfortable using one to learn about sensitive topics. All sessions were video and audio recorded to ensure accurate transcription and translation.

### Game development

The last decade has seen a significant increase in the use of digital games and game-like interventions to educate players and motivate behavior change ^9^,^10^. The use of games is particularly apt when seeking to reach young people given that play is still regularly a part of their lives. Play can provide a socially acceptable means of addressing topics, such as reproductive health, that may be difficult to discuss openly due to cultural taboos and stigmas ^11^,^12^. The *My Future Family* game is innovative in that it not only provides players with information, but simultaneously collects data about what matters to them and their future intentions.

Given that the students in the focus groups reported having limited access to computer and phone technology we determined that the best way to deliver the game was via tablets that could be taken into the schools. We ensured that all of the written text in the game was also spoken in English or the local language by the in-game narrator so students with a range of literacies would be able to understand. Headphones provided players with the relative privacy needed to be able to engage with sensitive material. The design metaphor for the game is a player walking through the valley of life, encountering 5 milestones: Become an Adult, Finish Schooling, Meet a Future Spouse, Get Married, Have Children. At each milestone, players unlock a mini-game that teaches them about puberty, intercourse, reproduction, and conception, respectively. At the beginning of each mini-game, players are asked to indicate the age at which they hope to reach the milestone and the main influencers on their decisions regarding the milestone. For example, a player could indicate that she wants to be 22 when she has her first child and that her mother and teacher are the most important influences on this decision. A video of gameplay and more information about the project are available at the game website. The game was built in Unity version 5.5.

### Game distribution and data collection

From August 1–17 2017, the research team piloted the game in 11 JSS rural and urban schools within a 200-kilometer radius of Mysore. 15 male and 15 female students aged 15–19 were randomly selected at each school and sent to a testing room. No names or id numbers were collected. Students were separated by gender to conform to cultural norms.

All in-game data was stored, including self-reported gender and age, age at which students desired to achieve each milestone, and time to complete mini-games. Upon completing the game, students were given a pencil-and-paper questionnaire in which they again reported demographic characteristics (age, gender, family structure), their desired number of children, and their perceptions of the game and how much they had learned. To validate the data collected in the game, it was compared with that collected in the questionnaires. Student gender was recorded on both the questionnaire and tablet by researchers prior to starting the game, to determine whether students played the game with avatars of a different gender.

In-game data and post-game questionnaires were analyzed and visualized using R version 3.4.3. All data used for analysis is publicly available at Open Science Framework. All code is available from GitHub.

### Ethics and consent

We obtained ethics committee approval from JSS Academy of Higher Education and Research (JSSMC/IEC/16/5525). The approval was reviewed by the IRB at Quinnipiac University. For the focus groups, written consent was obtained from all participants. All participants were informed that audio and videotapes of the focus groups would be stored in secure hard drive at the University of California Los Angeles’ secure database. They were reassured that neither audio nor videotapes would be used for any distribution other than research related activities. The institutional review board waived the written consent for the pilot testing of the game, as the risk was deemed minimal given the nature and anonymity of the game process. Audio and video files are stored electronically in secure drives. Actual DVDs of the video recording are stored in a locked cabinet. Data from the games are stored in the secure database in the form of electronic files. The data paper questionnaires were entered into the Redcap and actual questionnaires are stored in a locked cabinet at JSS Academy of Higher Education and Research.

## Results

### Focus groups

The principal findings gleaned from conversations with stakeholders were:

1. Adolescent reproductive health education: There was a clear paucity of structured sexual health education in schools and homes. The teachers reported that, at times, they educate girls about menstrual health and general health but avoid the topic of reproductive health. Parents did not want to discuss sexual matters at home. There was a general consensus among students that they wished to learn reproductive health at school, but not in a group setting where they could be embarrassed.
2. Fear of misuse of sexual health information: Parents and teachers alike had fear of the sexual health information being used to “get to the wrong path.” Adolescents did not share this concern.
3. Exposure to media, Internet, and cellphone: Across the board, rural and urban students had some access to cell phones. They had played games on phones and been exposed to the internet. Parents and teachers had concerns that students were obtaining sexual health information that was either skewed or beyond their level of understanding through media (TV/ads) and the internet.
4. Willingness to play a health education game: The adult participants in the focus groups encouraged the use of games to convey health information, and the students enthusiastically expressed a desire to learn sensitive information through gameplay. Parents and teachers did not seem to have any reservations about adolescents playing games for health.
5. Family planning values: We concluded that majority of the students in the focus group wanted 1–2 children without gender preference and desired relatively short (1–3 years) inter-pregnancy intervals.

### Gameplay (qualitative)

**Figure 1.**
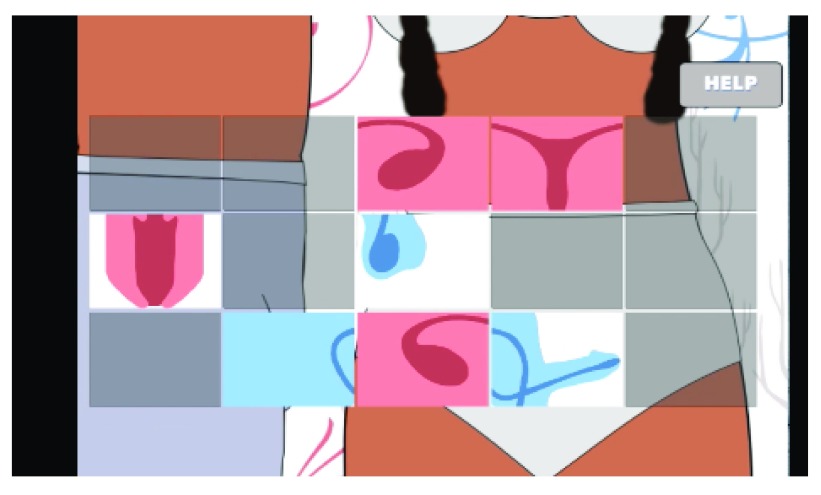
Screenshot 1: From the minigame attached to the Having Children milestone. Note the HELP button in the upper right.

 During game testing the majority of students were deeply engaged, often consulting their peers for help or suggestions on mini-games. Many players initially had difficulty understanding how to use the drag and drop interface, which required us to add a “help” button to certain minigames ( Figure 1; Screenshot 1) and give chalkboard-based tutorials prior to gameplay, but all of them were interacting with the game easily by the end of the session. We reached the following conclusions during this phase of the project:

1. If teachers remained in the room, the students approached the game as if it were a test that they were supposed to do well on. The research team wanted participants to have a freer experience of the game, so teachers were asked to leave.
2. Students had little or no experience using headphones which caused some technical difficulties. Headphones were integral to student’s experience of the game being private, which allowed them to approach sensitive material without embarrassment.
3. Paper questionnaires are not a good tool for gathering data from this population. We observed students having difficulty understanding questions, asking other students for help, and collaborating on answers. Written language and reading comprehension skills vary widely. For future iterations of the research project, pre and post-game data collection should be on the device that delivers the game using graphics and audio.
4. Students were universally enthusiastic about the game. During post-play debriefing, they expressed the desire that all the children in the school be able to play the game. Some suggested that it would be good for their parents to play as well. Participants also wanted more information from the game. They said that they wanted to learn about family planning methods and pregnancy.
5. The game interface allowed players to select to play either in English or in the local language (Kannada). Some of those who opted to play in English thinking that their language skills were good enough, were unable to understand some of the words and terms explaining reproduction and wanted to be able to switch to the other language which was not possible in the version of the game that was tested. Some players opted to restart the game so they could play in Kannada.

### Data collected from gameplay

There were 393 respondents overall, 200 male, 192 female, and one without reported gender. 15 game instances were excluded from analysis due to incomplete gameplay or absence of age/sex information.

**Figure 2.**
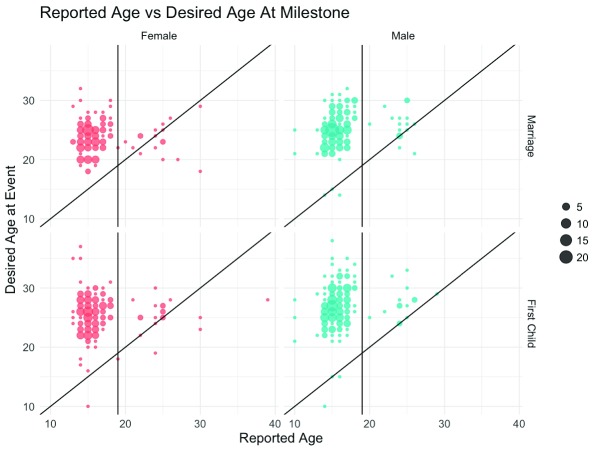
In-game reported age compared to age of milestones reported in the game, including line of equivalence (diagonal) and line marking age 19 (vertical).

96.3% of students self-reported the same gender during gameplay as was recorded prior to gameplay, showing that most students at least imagined an avatar of their own gender while planning out a fictional life course. Self-reported age was 16.3 on average but ranged from 10 to 39, a much broader spread than the target ages of 14–19. The self-reported age on the questionnaire, by contrast, had a much narrower range: from 13 to 19. This finding suggests that, although almost all players chose to play as avatars of their own gender, some opted to imagine avatars much older or younger than themselves. Figure 2 shows that the milestone responses for marriage and childbirth of those whose avatars are over 19 differ qualitatively from those who play with younger avatars. Teenaged avatars almost universally get married and have children at least 3–5 years later than their “current” age, and have virtually never “already” experienced these events. Older avatars, in contrast, tend to experience these milestones much closer to their current age, or sometimes many years in the “past”. Since the focus of this paper is to analyze students’ true life goals, we removed players who report an age younger than 13 or older than 19, or whose self-reported gender differed from their pre-recorded gender. This left a total sample size of 328 (167 male and 161 female).

**Figure 3.**
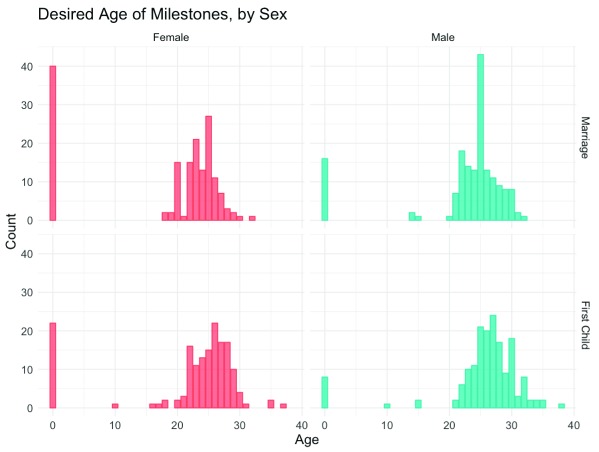
Desired age at marriage and first child milestones, by gender of respondent. The bars at age 0 indicate those who did not wish to fulfill that milestone.

The mean desired age at marriage was 24.9 (SD 2.94) for males and 23.7 (SD 2.54) for females, with 16 (9.6%) male and 40 (24.8%) female students stating that they did not want to get married. Even though the mean age at marriage was higher for males, the youngest desired age at marriage for female students was 18, whereas three male students listed their desired marriage age at 14 or 15 ( Figure 3). The mean desired age at first child was 26.8 (SD 3.59) for males and 25.4 (SD 3.44) for females, with 8 (4.8%) male and 22 (13.7%) female students stating that they did not want children.

**Figure 4.**
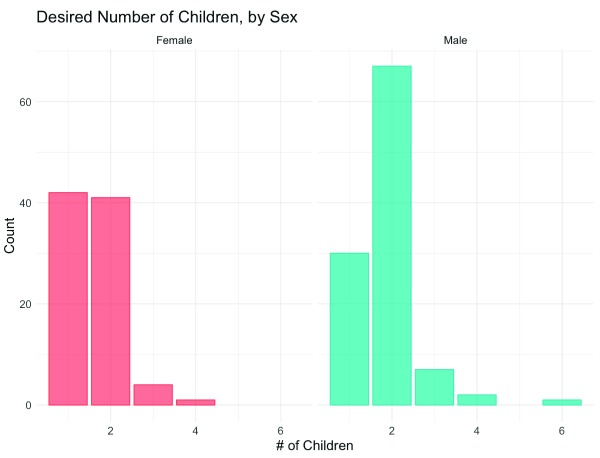
Total desired number of children, among those who specified a desired child count by gender of the respondent.

**Figure 5.**
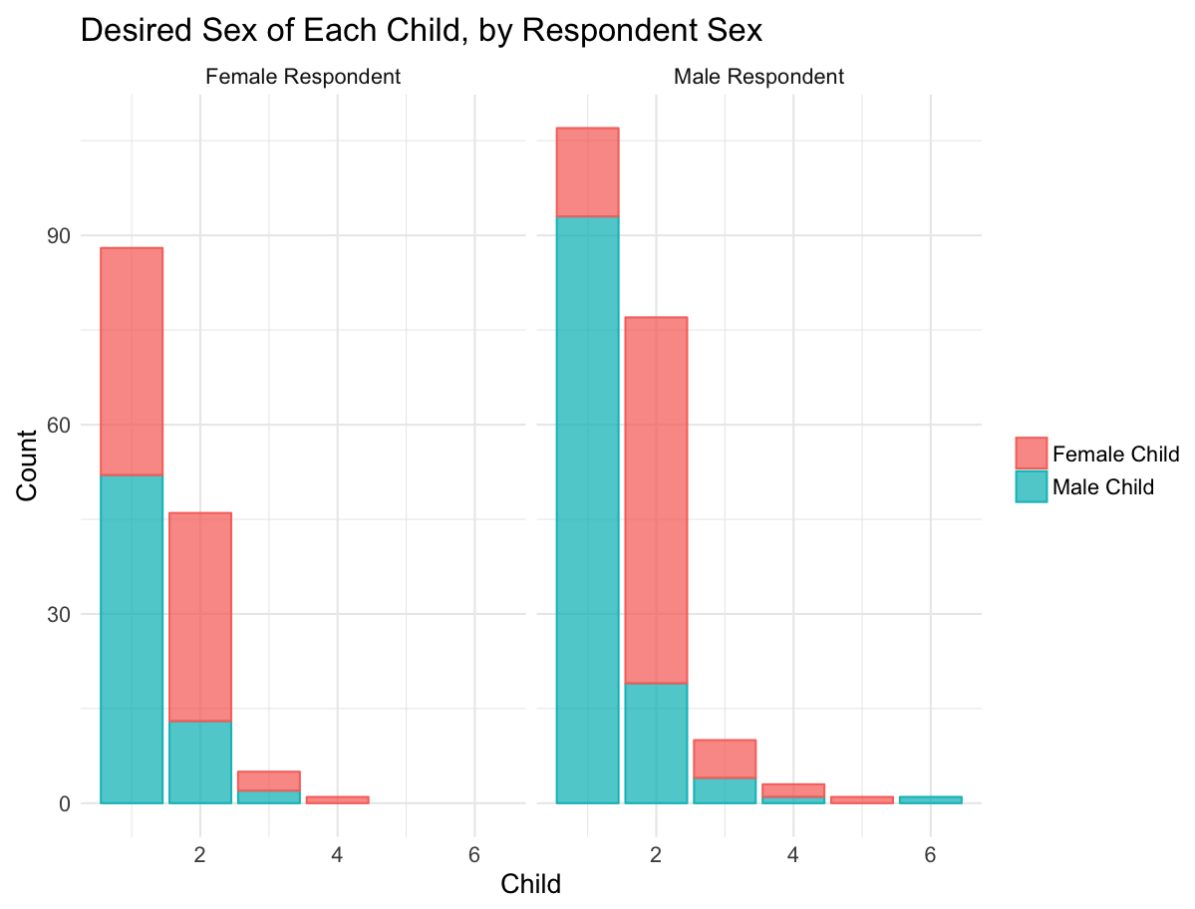
Desired sex of each child, by gender of the respondent.

Despite 298 respondents stating that they wanted children, only 195 (107 male and 88 female) explicitly stated the number, gender, and age at which they wanted these children ( Figure 4 and Figure 5). Female respondents wanted one or two children in approximately equal numbers (42 vs 41, 47.7 and 46.5%, respectively), while the majority of male respondents (62.6%) wanted two children. No female students wanted more than four children, whereas one male student wanted up to six.

The majority of both male and female students want their first child to be male and their second to be female, but both of these trends are more extreme for male respondents ( Figure 5)-- only 59% (52) of female respondents want their first child to be male, compared to 86.9% (93) of male respondents, while 75.3% and 71.7% of male and female respondents who want at least two children, want their second child to be female, respectively.

**Figure 6.**
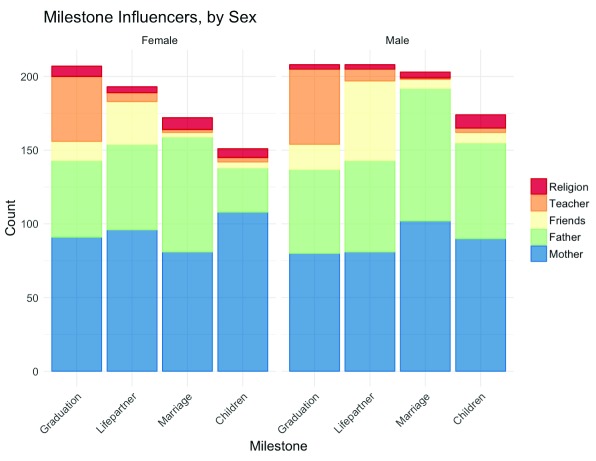
Reported “influencers” for each milestone, by gender.

For all milestones and both genders, students reported mothers and fathers as the primary influencers (mothers 38.5–71.5% of all listed influencers, fathers 19.9–45.4%), with teachers also playing a large role in the Graduation milestone (24.5% for males, 21.3% for females) and friends contributing heavily to the Life Partner milestone (26.0% for males, 15.0% for females, Figure 6). Fewer than 5.2% of students in any gender/milestone category reported religion as a key influencer. In Karnataka, mothers-in-law play a key influential role in many life decisions, but since no one in our dataset was married this figure does not appear in our list.

### Data from paper questionnaires

Post-game surveys gave students an opportunity to provide feedback on the quality and utility of the game, as well as state how much prior knowledge they had of the information delivered in the game.

Of the 382 students who took the post-game survey, 381 responded to the “Liked game” question, 379 to the “Would recommend game to friend” question, and 377 to the “Game improved knowledge” question.

**Figure 7.**
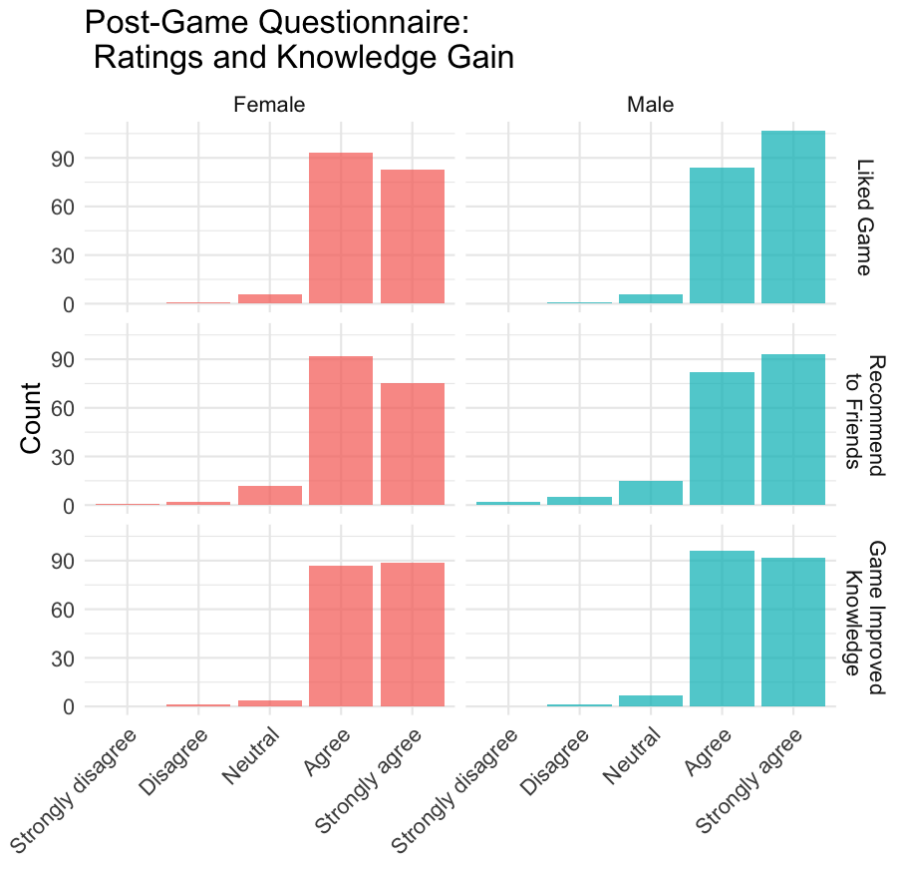
Quality-of-game responses from post-game questionnaire.

96.3% of respondents to the post-game survey strongly agreed or agreed that they enjoyed playing the game, and 90.2% strongly agreed or agreed that they would recommend it to their peers ( Figure 7). 96.6% strongly agreed or agreed that the game improved their knowledge about family planning, while 0.5% disagreed and none strongly disagreed.

**Figure 8.**
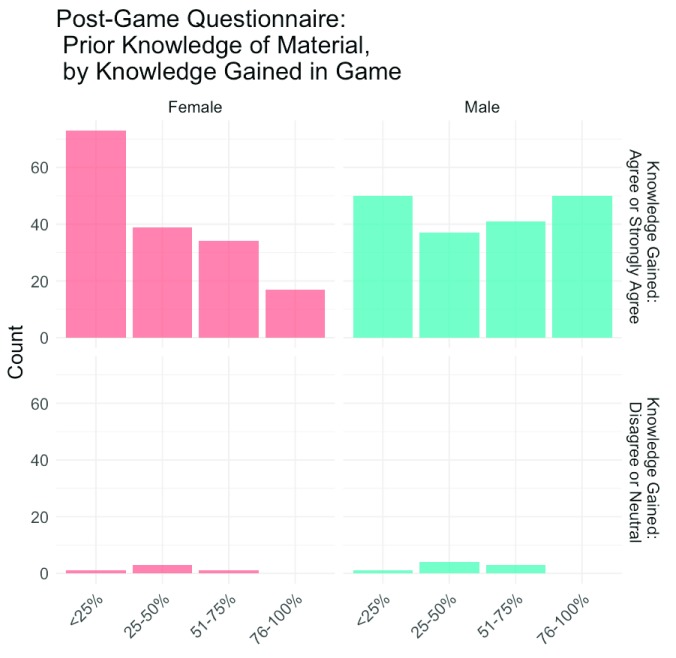
Perception of knowledge gained in game, by perception of knowledge already acquired.

When asked how much of the material in the game they already knew, 35.3% of 354 respondents reported knowing less than 25% of the in-game material, 23.4% reported knowing 25–50%, 22.3% reported knowing 50–75%, and 18.9% reported already knowing 75–100% of the in-game material ( Figure 8). Among the students (178 male, 163 female) who agreed or strongly agreed that the game improved their knowledge, trends in prior game knowledge differed. For female respondents, we found a clear negative relationship between prior knowledge and likelihood of “agreeing” or “strongly agreeing” to the “knowledge gained” question, while among male respondents there was no such trend ( Table 1).

**Table 1.** Distributions of respondents who agreed or strongly agreed to the “knowledge gained” question, by gender and self-reported prior knowledge.

	Male, % (N)	Female, % (N)	Total, % (N)
Prior Knowledge			
<25%	28.1 (50)	44.8 (73)	36.1 (123)
25–50%	20.8 (37)	23.9 (39)	22.3 (76)
51–75%	23.0 (41)	20.9 (34)	22.0 (75)
76–100%	28.1 (50)	10.4 (17)	19.6 (67)
Total	100 (178)	100 (163)	100 (341)

### Validation: Age at first child

**Figure 9.**
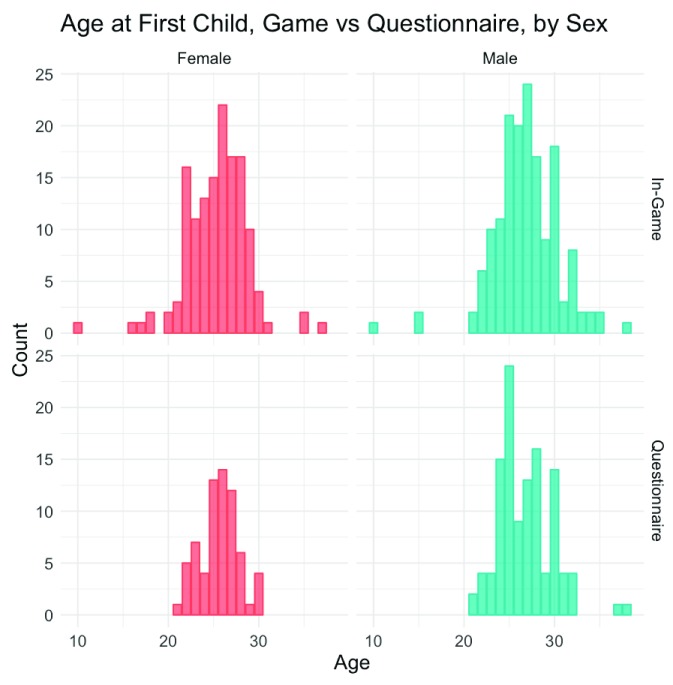
Distribution of desired age at first child within game compared to in post-game questionnaires.

The questionnaire also asked respondents at what age they would like to have their first child, giving us an opportunity to test how the in-game responses compared to the paper questionnaire to further test our hypothesis that students who play the game reporting their true gender and an age within 13–19 imagine their avatar as themselves. The mean age at first child listed in the questionnaire was 26.8 (range 21–38) for males and 25.6 (21–30) for females. These mean values are in almost-perfect agreement with the in-game ages of 26.8 (10–38) for males and 25.4 (10–37) for females, though the wider in-game ranges suggest some more speculative gameplay ( Figure 9). This high agreement suggests that, at least in populations similar to those surveyed, in-game responses may be used as effective proxies for formal family planning surveys.

## Discussion

The *My Future Family* game provided adolescents in urban and rural schools in the JSS Mahavidyapeetha school system in Karnataka with an engaging and stigma-free platform in which to learn basic reproductive health. Game development involved high levels of community engagement and buy-in from parents, students, and teachers. The game development team overestimated players’ proficiency with basic tablet interfaces. Students rated the game highly both in terms of playability and educational benefits, and repeatedly and enthusiastically requested more information. Data obtained from the game shows that, contrary to our expectations, young people in this area of India are well aware of the difficulty associated with large family size and do not need to be convinced to want fewer children. They want to have children when they are in their twenties and seek few and in some cases no children. They are well aware of their lack of knowledge of family planning and reproductive health and actively seek information on these topics.

We were concerned that there might be pushback from families or teachers who heard about the participants’ game experience following the pilot study. There were no reports of concern about the content of the game or participants’ experience from either parents or teachers.

Few participants listed religious figures as being strong influencers on decisions relating to family planning. The reasons for this could be explored further in future studies. The relative importance of parents suggests that interventions which seek to increase uptake of family planning methods should focus on delivering resources directly to future parents - as we did in this pilot by providing this information to young people who will be starting families soon.

This project showed the feasibility of utilizing interactive games to both educate and collect data from historically overlooked populations in developing countries. A game interface allows students to gain knowledge and comfort with a taboo topic in a relatively private space, and a non-stigmatizing game interface allows students to gain confidence in their knowledge without shame. Community engagement was key to this process-- had we built the game without focus groups or buy-in from local partners, we would have made a game whose goals did not line up with the objectives of our target audience.

### Limitations

While we received a qualitatively enthusiastic response from all stakeholders, we were not able to quantify knowledge gained by the game beyond self-reports. Without a longitudinal study, it is not possible to say what impact this game will have on students’ actual family planning choices. Limitations on literacy in the target population also limit the utility of paper questionnaires. The indicators analyzed suggested that students on the whole played the game as “themselves”, and that therefore in-game data can be interpreted as reflecting students’ actual desires and goals for family planning, but there may have been some students who played the game in a speculative manner that did not reflect their true plans. If the game is distributed in more urban areas where more students are already comfortable with technology and digital gameplay, this problem may be amplified.

Feedback on the game was overwhelmingly positive, but lack of negative responses may stem in part from cultural pressure to respect elders and not criticize the testing team. Both for collecting data and disseminating knowledge, future versions of the game will be rolled out to a much larger population, include pre- and post- game testing to directly assess knowledge gained, and include additional submodules on contraception and pregnancy.

### Next Steps

The results of the pilot provided the research team with important insights informing future developments re: a) how to improve the game b) the process for its distribution and dissemination, and c) measurement of outcomes and impact.

a) The interface for the game will be simplified so that players learn the drag and drop interface at the beginning of the game and the interface remains consistent throughout. Help buttons will be provided for each game so those who are unfamiliar with the material or interface can watch and learn. The game will allow players to switch between languages at any point in the game. New milestones will be added to provide information about family planning methods and pregnancy. The influencers will be expanded to include oneself on all milestones and parents-in-law on the Having Children milestone.

b) To have a significant impact, the game should be distributed to many more students than we were able to reach in the pilot. This is a challenge because much of the target population does not have access to a cellphone, tablet, or computer on which to play. We plan to partner with organizations in and outside of India who already have networks in place to achieve wider distribution. This will involve more focus groups to ensure that we maintain buy-in from all stakeholders. Ideally we would be able to demonstrate efficacy to governments and school administrators so that they would seek to distribute the game themselves.

c) Given that the goal of the game is to encourage young people to take an active role in planning their families and ultimately increasing uptake of safe and effective family planning measures, assessing impact of the game will require longitudinal studies that track how the behavior of adolescents who play the game differs from those who have not. We recognize the need to have shorter term measures that assess how playing the game impacts knowledge which we plan to achieve by building pre and post knowledge assessment into the game experience.

## Conclusion

We met our goal by developing a tablet-based game in which players provide information while playing minigames that provide detailed information about human sexual anatomy and reproduction through words, sound, and images. We found qualitative and quantitatively positive outcomes of gameplay, with students rating the game quality highly, recommending it to peers, and stating that they valued information learned from the game and wanted more. The quantity and quality of the data collected through this game demonstrate enormous potential for using it as a tool to increase understanding of this rising portion of the Indian population and as a means of providing them with the information they need to make informed choices about family planning in the future.

## Software availability

Further information including video play through of *My Future Family* can be found on the website: https://familyplanninggame.qu.edu/index.html

Source code for the *My Future Family* game can be found on GitHub: https://github.com/bertozzivill/india-family-planning

The game is available by request from the P.I. (EB)

## Data availability

The data underlying this study is available from Open Science Framework. Dataset 1: Collecting family planning intentions and providing reproductive health information using a tablet-based game in India.

DOI: http://doi.org/10.17605/OSF.IO/9YBJM ^13^

This dataset is available under a CC0 1.0 Universal license.

## Supplementary material

Supplementary File 1. Questions used in focus group meetings
