# Supplementary material for: Collecting family planning intentions and providing reproductive health information using a tablet-based video game in India
Source: Gates Open Res. 2018 Sep 7;2:20. Originally published 2018 Apr 26. [Version 2] doi: 10.12688/gatesopenres.12818.2 (PMC6030399; doi:10.12688/gatesopenres.12818.2)
Supplement: Supplementary file 1 [file gatesopenres-2-13937-s0000.tgz › bf5eb7f3-2466-4a53-85b4-8af6936e6d34.docx]

Supplementary File 1

Focus Group Questions

**Focus group for adolescents: FG1 and FG2:**

Introductory question:

a) Can you describe your ideal family (when you want to get married, to what type of person you want to get married to, do you want to have children, how many children and how far apart?)

- Each participant about 3 minutes (20 minutes)

b) What do you know about family creation? (how pregnancy occurs, how pregnancy can be prevented, should it be prevented) 3 minutes per participant (20 minutes)

c) Where do you get most of the information about this? (probe- if no answer family, school, friends, internet, phones, magazines) 1-2 minutes per participants (10 minutes)

d) Are you familiar with using phones and tablets? 1-2 minutes per participants (10 minutes)

Conclusion: What do you think about a game that you can play to explore these aspects? (7 minutes)

**II. Focus group for parents: FG2 and FG3:**

Introductory questions:

a) Do you talk to your child about future family planning?

- If yes- probe how
- If no- probe why not
- Each participant 5 minutes (30 minutes)

b) Do you think your children should know about family planning?

- If yes- probe how (probe how should that education be incorporated into their life)
- If no- probe why not
- Each participant 5 minutes (30 minutes)

c) Explain the overview of the game and open questions about what do they think of this project?

- Each participant 5 minutes (30 minutes)

d) What do you think about a game for teaching them family planning in the future?

- Each participant 5 minutes (30 minutes)

**III. Focus group for teachers: FG 5:**

Introductory questions:

a) Do you think your students should know about family planning?

- If yes- probe how (probe how should that education be incorporated into their life)
- If no- probe why not
- Each participant 5 minutes (30 minutes)

b) Explain the overview of the game and open questions about what do they think of this project?

- Each participant 5 minutes (30 minutes)

c) What do you think about a game for teaching them family planning in the future?

- Each participant 5 minutes (30 minutes)

d) What do we need to run a testing of game in your school?

- Each participant 5 minutes (30 minutes)
