# Supplementary material for: Collecting family planning intentions and providing reproductive health information using a tablet-based video game in India
Source: Gates Open Res. 2018 Sep 7;2:20. Originally published 2018 Apr 26. [Version 2] doi: 10.12688/gatesopenres.12818.2 (PMC6030399; doi:10.12688/gatesopenres.12818.2)
Supplement: Peer review history

# Peer review: Referee response for version 1

**DOI:** 10.21956/gatesopenres.13885.r26546

**Published:** 2018-06-28

**Authors:** Priya Nanda

**Affiliations:** 1 Bill & Melinda Gates Foundation, New Delhi, Delhi, India

This is an interesting study and even though it has a very limited sample size and no external validity, it still has important insights.  A few more domains could have been explored by the study.  The study should have shed more insights on inconsistent knowledge, more than it does.  Also it should tell us why people choose older avatars and the difference between those who did and who did not (were those who chose older avatars more uncomfortable being their own age and playing a game about sex?  Will design and content components differ in any way as a result of this study? Since the study was with such a small sample I wonder why authors did not collect more in-depth intervention to answer some of these whys.

I have read this submission. I believe that I have an appropriate level of expertise to confirm that it is of an acceptable scientific standard.

# Peer review: Referee response for version 1

**DOI:** 10.21956/gatesopenres.13885.r26461

**Published:** 2018-06-12

**Authors:** Sarah Averbach

**Affiliations:** 1 Department of Obstetrics, Gynecology, and Reproductive Science, University of California, San Diego, San Diego, CA, USA

This article describes the development and piloting of a tablet-based game which aims to provide adolescents with information about puberty, reproductive health, and family planning. The work is innovative and the manuscript is well written.  The manuscript includes qualitative results from focus groups and descriptive results from gameplay. The manuscript would be improved by expanding on the methods to ensure that the game is well understood to readers.

 Introduction:

 The introduction is concise and highlights the purpose of the research. The focus on population growth detracts from the objective to meet the reproductive needs of women and girls. It is not necessary to summarize results in the introduction.

 Methods:

 More information about the focus group participants would be helpful, how many participants per group, ages, genders, roles etc. Since gender identity was self-reported, the term “gender”, rather than “sex” should be used. Sex is the identify assigned at birth, based on assessment of external genitalia and sometimes chromosomes.

 What tools, if any, were adapted to create the curriculum for the game? For instance, when teaching adolescents about contraception are family planning methods reviewed individually? If so, in what order? Does the interactive platform allow for a person-centered approach to the teaching?

 Discussion:

 An additional limitation is that convenience sampling was used to select the 11 out of 126 schools where this study took place which could introduce selection bias.

 Next Steps:

 Future studies should focus on evaluating impact of the game on reproductive health choices.

 If a large longitudinal study is planned to measure the effect of the game on family planning choices, a careful dissemination and implementation framework should be developed concurrently.

I have read this submission. I believe that I have an appropriate level of expertise to confirm that it is of an acceptable scientific standard.
